# Ordinal classification of the affectation level of 3D-images in Parkinson diseases

**DOI:** 10.1038/s41598-021-86538-y

**Published:** 2021-03-29

**Authors:** Antonio M. Durán-Rosal, Julio Camacho-Cañamón, Pedro Antonio Gutiérrez, Maria Victoria Guiote Moreno, Ester Rodríguez-Cáceres, Juan Antonio Vallejo Casas, César Hervás-Martínez

**Affiliations:** 1 Department of Quantitative Methods, Universidad Loyola Andalucía, Córdoba, Spain; 2 Department of Computer Science and Numerical Analysis, University of Córdoba, Córdoba, Spain; 3 UGC Medicina Nuclear, Hospital Universitario “Reina Sofía”, IMIBIC, University of Córdoba, Córdoba, Spain; 4 Provincial TICS Team, Hospital Universitario “Reina Sofía”, IMIBIC, University of Córdoba, Córdoba, Spain

**Keywords:** Parkinson's disease, Computer science, Statistics

## Abstract

Parkinson’s disease is characterised by a decrease in the density of presynaptic dopamine transporters in the striatum. Frequently, the corresponding diagnosis is performed using a qualitative analysis of the 3D-images obtained after the administration of $^{123}$I-ioflupane, considering a binary classification problem (absence or existence of Parkinson’s disease). In this work, we propose a new methodology for classifying this kind of images in three classes depending on the level of severity of the disease in the image. To tackle this problem, we use an ordinal classifier given the natural order of the class labels. A novel strategy to perform feature selection is developed because of the large number of voxels in the image, and a method for generating synthetic images is proposed to improve the quality of the classifier. The methodology is tested on 434 studies conducted between September 2015 and January 2019, divided into three groups: 271 without alteration of the presynaptic nigrostriatal pathway, 73 with a slight alteration and 90 with severe alteration. Results confirm that the methodology improves the state-of-the-art algorithms, and that it is able to find informative voxels outside the standard regions of interest used for this problem. The differences are assessed by statistical tests which show that the proposed image ordinal classification could be considered as a decision support system in medicine.

## Introduction

Parkinson’s disease (PD) is a progressive, neurodegenerative disease that causes characteristic motor symptoms of tremor, bradykinesia, and postural instability^1^. PD is caused by deterioration of the dopaminergic neurons in the extrapyramidal tract of the midbrain, that modulates voluntary movements and controls maintenance of posture and coordination of gait. Degeneration of neurons that release dopamine causes an imbalance of excitatory (acetylcholine) and inhibitory (dopamine) neurotransmitters in the region^2^.

Presynaptic dopamine transporter density can be detected by neuroimaging techniques, which are now standard practice in the diagnosis of neurodegenerative disorders such as PD. Dopamine deficiency in the striatum can be evaluated using nuclear medicine techniques. The $^{123}$I-ioflupane (DaTSCAN, General Electrics Healthcare Limited, Little Chalfont. Bucks HP79NA U.K.) is a radiopharmaceutical, widely used for this purpose, which binds to the presynaptic dopamine transporters in the caudate and putamen and allows the density of these to be evaluated with high sensitivity^3,4^. This method enables an early diagnosis of neurodegenerative parkinsonism. DaTSCAN can be suitable for assessing the presynaptic deficit in early stages of disease. Moreover, it can differentiate patients with neurodegenerative parkisonism from those with other forms of parkinsonism^5^.

Visual assessment allows evaluating the normality of dopamine transporter (DAT) binding and the magnitude of compromised DAT binding, specially focusing on asymmetry and affected structures^6^. In clinical practice, DATSCAN images are commonly interpreted with careful visual assessment of the striatal tracer binding. This approach has high diagnostic accuracy and excellent interobserver agreement^7^. But this visual classification may be subjective and strongly depends on the experience and the fatigue of the person in charge of the labelling. Also, binding quantification is based on a manual delineation of striatal regions of interest (ROIs), but this technique is still subjective and dependent on one operator^8^.

Currently, visual diagnoses, with one or several specialised observers, are combined with automatic computer systems that analyse the data and are able to distinguish between two classes, pathological or normal. In the case of PD diagnosis, the problem was initially tackled with a classical approach, i.e. by quantifying the loss of neuronal dopamine in the striatum^9^. Posterior attempts to automatise PD diagnosis used semi-quantitative assessment of images with $^{123}$I-Ioflupane^10,11^. Moreover, computer systems based on machine learning^12^ has recently shown promising results based in the striatal size and shape and on different image properties. In general, most advanced computational techniques of examining images could help identify the disease and build effective decision-support systems for the diagnosis of Parkinson. The European Association of Nuclear Medicine (EANM) recommends quantitative analysis, associated with this type of support models^6^.

The majority of works that seek to address the problem of classification of pathological and normal patients, based on their functional images, apply automatic learning techniques based on ROIs^13^. Using the semi-quantitative methods recommended by the EANM guidelines, independent ROIs of caudate, putamen and occipital background of the axial cuts that integrate the grooves are performed, and the relationship between the different regions is analysed. However, training binary classification models (pathological and normal) by treating these ROIs as the units of information ignores voxels outside the ROIs and can limit performance. Note that a random variable associated with a voxel (minimum volumetric unit of the image) can sometimes be significantly important for the classification task, so it is important to treat them individually. In this way, specific voxels, within the region, may have greater dopamine transporter loss than others and therefore may be more informative. Direct relations between the voxels and the classification task can be found and, by ignoring these ROIs, we avoid letting ourselves be guided by prior medical influence.

Extra-striatal regions may contain a significant amount of $^{123}$I-ioflupane dopamine targets, and the use of these regions may improve the statistical reliability of the model. This could be the case, for example, if extra-striatal regions are combined with the caudate to provide a reference for comparison with the putamen. Including all voxels in these regions as discriminant characteristics and letting a feature selection algorithm decide which voxels are more informative (i.e. informative voxels) seems to have a higher potential than assuming *a priori* which voxels are important according to some expert knowledge. Therefore, our study develops an algorithm based on voxels rather than based on classical ROIs. In studies with 18F-fluorodeoxyglucose PET, increases of metabolic activity were identified in pallidothalamic and pontine areas^14^. Recent experimental studies, using a NMR-based metabolomic approach, show metabolic imbalance among different brain regions, especially in the midbrain and right cortex^15^.

In this work, SPECT 3D-images obtained after the administration of $^{123}$I-ioflupane are labelled in three classes depending on the level of affectation of the image, that is, without alteration of the presynaptic nigrostriatal pathway (class 0), with a slight alteration (class 1) or with severe alteration (class 2)^16^. In this sense, and given the natural order between classes ($0<1<2$), we propose to use ordinal classification models. The main objective is not only a correct classification of the patterns but also a nerror reduction for the misclassified ones. In other words, if the classifier cannot correctly classify a pattern of the class 0, the misclassification in class 1 is preferred than an error in class 2. This paradigm has been applied in a lot of prediction problems of medicine^17^, such as cesarean section rates^18^, breast cancer^19^, liver transplantation^20^, or Alzheimer progression^21^, among others. Therefore, the application of ordinal classification to PD diagnosis seems to be appealing.

Grading in PD could be defined as the act of classifying patients according to a global staging or severity ranking. New formulations of levodopa and novel delivery systems are currently being evaluated and gradually introduced in clinical practice in an attempt to prevent or treat levodopa-related motor complications. With this gradation, we could study when is the best moment to introduce levodopa or other treatments in the future^22^. Furthermore, this gradation is relevant for several reasons as, for example, allowing analysis of the relationships between group’s characteristics (grades or levels) and many other factors (duration of disease, effects of therapy, etc.); intergroup comparison, selection of patients for clinical research...^23^. Pasquini et al.^24^ also suggest that caudate quantification of DAT availability shortly after diagnosis may have an important role in identifying patients at risk of clinical progression to cognitive impairment, depression and gait problems in the near future. We can use the scales for outcome in Parkinson’s Disease–Motor (SCOPA-Motor)^25^ or the Non-Motor Symptoms Scale (NMSS). The NMSS has 30 items in nine domains^26^. For the purpose of obtaining an objective quantification technique, more complicated methods need to be proposed, and observer-independent automated quantification methods are preferable^27^.

Given the large number of features presented in these 3D-images, and considering all the image voxels (instead of putamen and caudate) as we stated ahead, it is necessary to use techniques to reduce them. It is one of the problems tackled in this work. It is true that, in machine learning, there are several techniques for the reduction of the number of features, for instance, compressive sensing-enhanced feature selection^28^ or using evolutionary computation^29^. The methods of machine learning for the selection of characteristics can, in general, be divided into two classes: wrapper^30^, filter^31^, and embedded methods^32^. In this paper, we will use a filter method where each feature is ranked individually based on specific statistical measures without taking into account the type of learning algorithm used in the classifier. We will use the ReliefF algorithm as a feature selector for a nominal classifier^33,34^, to then develop for ordinal classification. ReliefF makes a ranking of the features according to their discriminatory quality, where the user needs to specify the percentage of the best characteristics to be selected.

Another problem that we need to tackle is the low number of patterns of the dataset for the least frequent class (slight alteration). The most natural and most common method to reduce overfitting on image data is to artificially enlarge the dataset using label-preserving transformations^35,36^. A common practice for augmenting datasets is padding 4 pixels on each side and then doing random cropping and random flipping on the fly during training^37^. In the same way, in ImageNet dataset^38^, it is common to subtract the mean and divide by the standard deviation for each input channel and follow the data augmentation as described by Krizhevsky et al.^39^ In the context of imbalanced classification, the noisy replication method^40^ has been proven to be an effective approach in improving accuracy for the minority classes, specially for binary classification problems. It randomly chooses minority patterns and replicate them adding a small amount of noise. In this work, we develop a new method to generate new synthetic images and voxels based on the adjustment of the statistical distribution of the voxels, where, given that the same coordinate system is used for all 3D images, we can unequivocally characterise the position of a given voxel.

There are some recent approaches that consider machine learning and advanced computational techniques for the detection of PD. It is common to the use of collected voice measures. For instance, Kaur et al.^41^ proposed an ensemble of 25 state-of-the-art regression models to analyse the prediction of the motor Unified Parkinson’s Disease Rating Score (UPDRS). The number of patients (42) is considerably limited. Canturk^42^ considers a combination of the dynamic spiral test (DST) and static spiral test (SST) for early detection of PD. For this, the fuzzy recurrence plot is used to transform time series into grayscale images, which are then analysed using two deep networks (AlexNet and GoogleNet). The dataset used is based on 62 PD patients and 15 healthy ones. The conclusions are that the Y signal provides better results for DST, while a combination of Y and P signals performs better in SST. In the work proposed by Naseer et al.^43^, deep transfer learning is applied to the identification of PD, using as input handwriting images, which are one of the earliest indicators of the affection. The source tasks are ImageNet and MNIST datasets, and the use of freeze and fine-tuning of transfer learning is investigated in combination with data augmentation. Based on a dataset of 37 PD patients and 38 healthy subjects, the best results are finally obtained with fine-tuning-based approach considering the ImageNet and PaHaW dataset. However, all these works approach the problem as a binary classification problem (considering whether the disease is present or not) and none of them uses 3D-images of $^{123}$I-ioflupane as input data.

The main objective of this work is to obtain a tool which acts as decision support system in PD diagnosis for assessing the level of affectation of the patients using 3D images. The use of grading system (instead of a binary classifier) would increase the information obtained by the experts to better decide the treatment. Moreover, as the complexity of the problem increases (three classes instead of two), another objective is to include specific methods to deal with the imbalance ordinal nature of the dataset. Summarising, the main contributions of this paper are:

- The use of an ordinal classifier due to the natural order of the labels in the dataset. The patterns will be classified into three classes: no alteration of the pathway (class 0), slight alteration (class 1) or severe (class 2) alteration. For that, we use the ordinal logistic regression model available in the software *mord* developed by Pedregosa-Izquierdo^44^.
- The development of a new method to reduce the number of characteristics of an ordinal classification dataset. The method, referred to as ordinal ReliefF, is a modification of the standard state-of-the-art ReliefF^34^, taking the ordinal nature of the problem into account.
- The development of a new technique for generating synthetic patterns based on the statistical distribution of the macrovoxels. This method tries to find the best statistical distribution for each voxel (selected from a set of well-known distributions). Then, new voxels are generated using this distribution. The synthetic image will be used as part of the training set to improve the quality of the classifier.
- The application of the methodology into a real-world dataset obtained by the UGC Medicina Nuclear of the Hospital Universitario “Reina Sofía” (Córdoba, Spain), including 434 studies with different levels of alteration of the presynaptic nigrostriatal pathway: 271 without alteration, 73 with a slight alteration, and 90 with severe alteration.

The rest of paper is organised as follows: “Methodology” section presents the details of the proposed method. “Dataset and experimental design” section describes the data considered and the characteristics of the experiments, while “Results and discussion” section includes the results and the associated discussion. Finally, “Conclusion” section concludes the paper.

## Methodology

As we stated before, the goal is to create an automatic method that classifies the 3D-images into three ordered classes corresponding to: no alteration of the presynaptic nigrostriatal pathway (both striatum were visually conserved), with a slight alteration (just one putamen was altered), and with a severe alteration (alteration in both). As can be seen, it results in an ordinal classification problem, given that, for example, the error when classifying a severe alternation patient as no alteration is not the same as categorising it in the slight alteration class.

An ordinal classification problem consists in the prediction of the label *y* of a given input vector ${\mathbf {x}}$, where ${\mathbf {x}} \in {\mathscr {X}} \subseteq {\mathbb {R}}^S$ and $y \in {\mathscr {Y}} = \{{\mathscr {C}}_1, {\mathscr {C}}_2, \dots , {\mathscr {C}}_L\}$, i.e. the input vector ${\mathbf {x}}$ is in a *S*-dimensional input space and *y* is in a label space of *L* different possibilities. Given a training set of *N* patterns, defined as $D = \{({\mathbf {x}}_i,y_{{\mathbf {x}}_i}), i=1,\dots ,N\}$, the objective is to find a classification rule or function $f: {\mathscr {X}} \rightarrow {\mathscr {Y}}$ to predict the categories of new patterns. It is important to mention that a natural order is found in the labels of an ordinal classification problem, that is, ${\mathscr {C}}_1 \prec {\mathscr {C}}_2 \prec \dots \prec {\mathscr {C}}_L$, where $\prec$ is an order relation. It makes that two different elements of ${\mathscr {Y}}$ could always be compared using the relation $\prec$, which is not possible in nominal classification. However, labels in ordinal classification do not carry metric information (we can not establish the distance between the categories), and the category serves more as a qualitative indication.

According to the taxonomy proposed by Gutierrez et al.^45^, there are three groups of ordinal classification methods. The first one is called *naïve approaches*, which derive the model by using other standard procedures (such as regression or nominal classification). The second one is related to *ordinal binary decomposition approaches*, where the main idea is to decompose the ordinal problem into several binary ones. And the third one is the set of methods known as *threshold models*, which are based on approximating a real value predictor and then dividing the real line into intervals.

This work is focused on the third group. Methods within this group estimate a function $f({\mathbf {x}})$ for the prediction of the values of the output variable, and a set of thresholds ${\mathbf {b}} = (b_1,b_2,\dots ,b_{L-1}) \in {\mathbb {R}}^{L-1}$ to represent intervals in the range of $f({\mathbf {x}})$ which must satisfy the constraints $b_1 \le b_2 \le \dots \le b_{L-1}$. In this context, cumulative link models extend the binary logistic regression to ordinal classification by predicting probabilities of groups of continuous categories taking the ordinal scale into account, that is, cumulative probabilities $P(y\preceq {\mathscr {C}}_j|{\mathbf {x}})$. In this work, we use an interesting alternative called immediate-threshold approach^46^. It is based on including $L-1$ thresholds partitioning the real line to *L* segments and on penalising the predictors outside the correct segment or too close to its edges, considering, for each labelled example, only the two thresholds limiting this segment.

For the comparison of methods in ordinal classification we should take into account the following considerations. Ordinal classification problems must be evaluated with specific metrics. At first sight, various measures of ordinal association and product–moment correlation and regression appear to be based on very different foundations.

In this work, we have used metrics based on a product-moment system. In this system of metrics, the ones most commonly considered in machine learning for ordinal classification are (1) the mean absolute error (here denoted as *MAE*)^47,48^, also called classification loss^49,50^. The *MAE* is defined as the mean deviation of the predicted class from the true class (expressed both as integers). (2) The mean zero-one error (*MZOE*, more often referred to as error rate)^48^, where $MZOE=1-CCR$, where *CCR* is the correct classification rate or accuracy.

Unlike *MAE*, *MZOE* has the disadvantage that all errors are treated equally, so it does not sufficiently penalise algorithms that make errors of more than one class in the ordinal scale (e.g. severe alterations classified as no alteration). This kind of errors are penalised by *MAE*. However, these measures are not adequate when used to evaluate the performance of classifiers on imbalanced ordinal datasets^47^.

Given that, in our work, some of the classes have a much lower number of patterns than the others (there is a lower number of patients with alterations), we propose to use the maximum *MAE* metric (here denoted as *MMAE*)^51^, which measures the performance in the worst ranked class.

In contrast to most previous works that try to obtain a high global accuracy in the test set, we try to achieve a high level of classification rate with a good level of classification for each individual class. In this way, the pair of metrics including *CCR* and *MMAE* evaluates two characteristics associated with a classifier: the overall performance and the average deviation of the worst classified class. As theoretically demonstrated in previous studies^51,52^, these two objectives, above certain levels, are conflicting during an optimisation process, i.e. increasing one of the metrics can be achieved by worsening the other (e.g. we can trivially increase *CCR* by classifying almost all patterns in the no-alteration class, which will drastically worsen *MMAE*, given the imbalance character of the dataset). Next, we formally define both metrics.

On the one hand, *CCR* is the percentage of patterns correctly classified and is defined by:

1 $$\begin{aligned} CCR = \frac{1}{N} \sum _{i=1}^N I(y_{{\mathbf {x}}_i}={\hat{y}}_{{\mathbf {x}}_i}), \end{aligned}$$

where ${\hat{y}}_{{\mathbf {x}}_i}$ is the target predicted for ${\mathbf {x}}_i$, and $I(y_{{\mathbf {x}}_i}={\hat{y}}_{{\mathbf {x}}_i})$ is the indicator function.

On the other hand, the *MAE* is a measure of error that takes into account the ordinality of the target variable:

2 $$\begin{aligned} MAE = \frac{1}{N}\sum ^{N}_{i=1}|y_{{\mathbf {x}}_i}-{\hat{y}}_{{\mathbf {x}}_i}|, \end{aligned}$$

where $|y_{{\mathbf {x}}_i}-{\hat{y}}_{{\mathbf {x}}_i}|$ is the absolute distance between the actual and predicted labels. *MAE* ranges from 0 to $L-1$ (which is the maximum deviation in number of categories). However, in imbalanced problems, the most frequent classes can dominate the *MAE* error, masking poor performance for less common classes. That is the reason why *MMAE* is defined as follows:

3 $$\begin{aligned} MMAE = \max \left\{ MAE_1,MAE_2,\ldots ,MAE_l,\dots ,MAE_L\right\} , \end{aligned}$$

where $MAE_l$ is the *MAE* error taking into account only patterns from class *l*:

4 $$\begin{aligned} MAE_l = \frac{1}{N_l}\sum ^{N_l}_{i=1}|y_{{\mathbf {x}}_i}-{\hat{y}}_{{\mathbf {x}}_i}|, l=1,\dots ,L,. \end{aligned}$$

The following subsections present the proposed methodology which is divided into three phases. The first one corresponds with the preprocessing of the 3D-images, which includes a spatial normalisation and the transformation from 3D-images into 1D-arrays. The second one consists in the reduction of the number of characteristics. And finally, the last one is the application of a technique of data augmentation using the probabilistic distribution of the macrovoxels.

### Preprocessing of the SPECT 3D-images

The SPECT images can be obtained in different conditions, for example, due to the inclination or rotation of the patient during the taking of the image, two 3D images may look different, but they are really the same, with different orientation. To solve this problem, we use the open source software Platform for the Evaluation of Medical Imaging (PETRA)^53^. PETRA is a toolbox developed to analyse neuroimaging data using multivariate techniques, specifically designed to work with PET, SPECT, etc. images with the goal of Alzheimer and Parkinson diseases diagnosis/detection. This software has been used to perform a spatial normalisation, i.e. to align all brains in the same spatial disposition, using the same reference number to give the same coordinates to all voxels regardless of the brain.

**Figure 1:**
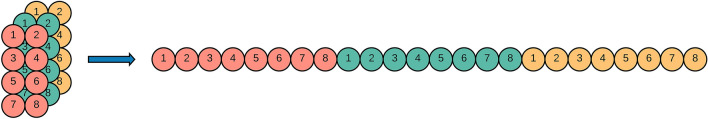
Example of a 3D-image of size $4\times 2 \times 3$ reshaped to a 1D-array.

Once the spatial normalisation is done, the 3D-images are converted into a one-dimensional array to work with them for the next operations. Figure 1 shows the procedure for a toy 3D-image of size $4\times 2\times 3$ (rows $\times$ columns $\times$ layers).

### Feature selection: ReliefF and proposed ordinal ReliefF

Given the large amount of voxels present in SPECT images (see “Dataset and experimental design” section for more details on the size of the images used in this paper), and considering that we use all image voxels instead of the ROIs, we need to reduce the number of features using automatic machine learning techniques.

For that, as we stated in the introduction, we initially use a filter feature selector in such a way that this selection does not take into account the learning algorithm used by the classifier. This filter, which is called ReliefF^34^, is based on ranking the features using the quality of them according to how well their values distinguish between instances that are near to each other. For that, the quality, *Q*, of each attribute, *A*, is initialised to $Q[A]=0$, and then, the following procedure is repeated *m* times (usually, *m* is set to the number of instances *N*):

1. Randomly select an instance, a 1D-array of the 3D-image in our case, ${\mathbf {x}}_i$.
2. Find the *k* nearest neighbours of the same class, called nearest hits: ${\mathbf {x}}^*=\{{\mathbf {x}}_{1}^*,\dots ,{\mathbf {x}}_{k}^*\}$, where $\forall j \in \{1,\dots ,k\}$, ${\mathbf {x}}^*_{j} \in {\mathscr {C}}_{{\mathbf {x}}_i}$ and ${\mathbf {x}}_i\ne {\mathbf {x}}^*_{j}$. ${\mathscr {C}}_{{\mathbf {x}}_i}$ is the class which includes ${\mathbf {x}}_i$.
3. For each class $${\mathscr {C}}_l\ne {\mathscr {C}}_{{\mathbf {x}}_i}$$, find the *k* nearest neighbours, called nearest misses: $${\mathbf {x}}^{**}=\{{\mathbf {x}}_{1l}^{**},\dots ,{\mathbf {x}}_{kl}^{**}\}$$, where $$\forall j \in \{1,\dots ,k\}$$, $${\mathbf {x}}^{**}_{jl} \in {\mathscr {C}}_{l}$$.
4. For each attribute *A*, we update its quality *Q*[*A*] taking into account that different values of the attribute *A* for instances in the same class will decrease the quality estimation *Q*[*A*], while different values for instances in different classes, which is desirable, will increase the quality *Q*[*A*]. The following expression updates the *Q*[*A*] values:
  5 $$\begin{aligned} Q[A] = S Q[A] - \frac{\sum _{j=1}^k \text {diff}(A,{\mathbf {x}}_i,{\mathbf {x}}^*_j)}{k} + \frac{\sum _{{\mathscr {C}}_l\ne {\mathscr {C}}_{{\mathbf {x}}_i}} \left[ \frac{P({\mathscr {C}}_l)}{1-P({\mathscr {C}}_{{\mathbf {x}}_i})} \sum _{j=1}^k \text {diff}(A,{\mathbf {x}}_i,{\mathbf {x}}^{**}_{jl}) \right] }{k}, \end{aligned}$$
  where $P({\mathscr {C}}_l)$ is the prior probability of class ${\mathscr {C}}_l$ which is estimated from the frequency of class ${\mathscr {C}}_l$ in the training set, and diff$(A,{\mathbf {x}}_1,{\mathbf {x}}_2)$ is defined as:
  6 $$\begin{aligned} \text {diff}(A,{\mathbf {x}}_1,{\mathbf {x}}_2) = |value(A,{\mathbf {x}}_1) - value(A,{\mathbf {x}}_2)|, \end{aligned}$$
  where $value(A,{\mathbf {x}}_i)$ is the value of the attribute *A* for the instance ${\mathbf {x}}_i$. This function is also used to calculate the distance between two instances (to determine the *k* nearest neighbours), where the total distance is the sum of distances over all attributes (Manhattan distance).

In this work, we propose a novel variation of the ReliefF, called ordinal ReliefF (OReliefF), which takes the natural order of the labels of the dataset into account. The modification consists in the inclusion of an ordinal penalty ($\rho _{{\mathscr {C}}_l}$) in the second term of the equation:

$$\begin{aligned} Q[A]&= Q[A] - \frac{\sum _{j=1}^k \text {diff}(A,{\mathbf {x}}_i,{\mathbf {x}}^*_j)}{k} \\&\quad + \frac{\sum _{{\mathscr {C}}_l\ne {\mathscr {C}}_{{\mathbf {x}}_i}} \left[ \frac{P({\mathscr {C}}_l)}{1-P({\mathscr {C}}_{{\mathbf {x}}_i})} \rho _{{\mathscr {C}}_l} \sum _{j=1}^k \text {diff}(A,{\mathbf {x}}_i,{\mathbf {x}}^{**}_{jl}) \right] }{k}, \end{aligned}$$

where $\rho _{{\mathscr {C}}_l}$ is defined as:

7 $$\begin{aligned} \rho _{{\mathscr {C}}_l} = \frac{|y_{{\mathbf {x}}_i} - y_{{\mathscr {C}}_l}|}{\sum _{{\mathscr {C}}_l^{'}\ne {\mathscr {C}}_{{\mathbf {x}}_i}}|y_{{\mathbf {x}}_i} - y_{{\mathscr {C}}_l^{'}}|}, \end{aligned}$$

where $y_{{\mathbf {x}}_i}$ is the class label of the pattern ${\mathbf {x}}_i$, $y_{{\mathscr {C}}_l}$ is the label of the class ${\mathscr {C}}_l$, and $y_{{\mathscr {C}}_l^{'}}$ is the class label of ${\mathscr {C}}_l^{'}$.

As can be seen, with $\rho _{{\mathscr {C}}_l}$, OReliefF penalises less the differences of those patterns which are in a class near to the class of pattern ${\mathbf {x}}_i$ (${\mathscr {C}}_{{\mathbf {x}}_i}$), giving more importance to different values for instances in farthest classes.

### Data augmentation

Given the difficulty of obtaining SPECT 3D-images for different reasons such as patient privacy, the high cost of taking images or the necessity to carry out inter-centre studies due to the different hardware and software used in different nuclear medicine units, we develop new methods to generate synthetic images for the training set in order to improve the quality of the classifier. For this, we propose a methodology based on the generation of new 3D-images using the probabilistic distribution of the macrovoxels. The algorithm 1 summarises this procedure.

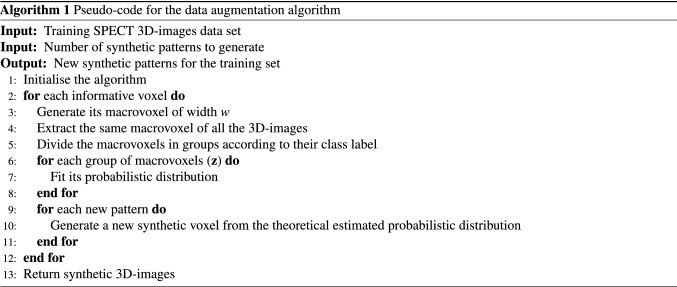



**Figure 2:**
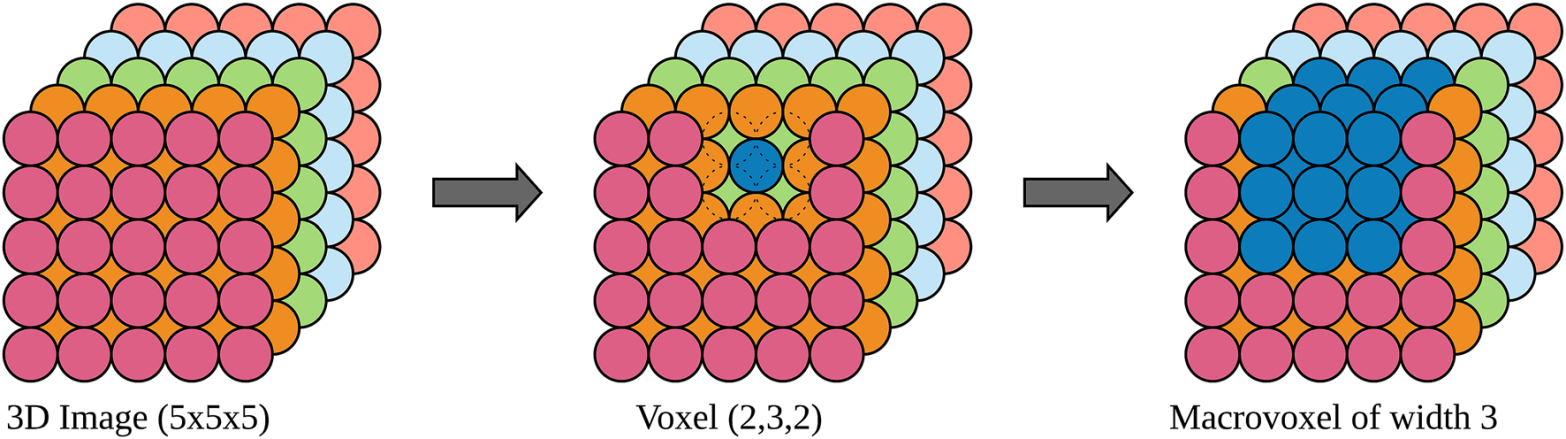
Macrovoxel of width 3 of the voxel (2,3,2) in a 3D-image of size $5\times 5\times 5$.

As can be seen, for each informative voxel determined by the OReliefF algorithm, we select its macrovoxel which is defined as the cube of voxels of width *w* where the selected voxel is in the middle. For example, Fig. 2 shows an image of dimensionality $5\times 5\times 5$, where the macrovoxel of width 3 based on voxel (2,3,2) ($row=2, col=3, layer=2$) is the one which is coloured in blue.

After that, the same macrovoxel is extracted for each 3D-image *i* and, for each class, all the macrovoxels of the different 3D-images are grouped. In this way, for each voxel, we have *L* groups of macrovoxels corresponding to each class label, so each group is formed by all the voxels inside all these selected macrovoxels.

These groups (${\mathbf {z}}$) are use to calculate the probabilistic distribution of the voxel. To do so, we consider a set of possible probabilistic distributions. These distributions has been selected due to the fact that they are the best-fitted ones in almost all voxel groups considering the distributions presented in the scipy library (see https://docs.scipy.org/doc/scipy/reference/stats.html). These are:

- **Alpha** distribution, whose probability density function (pdf) is:
  8 $$\begin{aligned} f(z,\alpha ,\mu ,\sigma ) =&\frac{1}{((z-\mu )/\sigma )^2\Phi (\alpha )\sqrt{2\pi }} \cdot \exp (-\frac{1}{2}(\alpha - \frac{1}{((z-\mu )/\sigma )})^2), \end{aligned}$$
  where $\Phi$ is the normal cumulative distribution function (CDF), $\mu$ and $\sigma$ are the location and scale parameters, respectively, which are considered in all distributions, and $\alpha$ is the shape parameter.
- **Generalised extreme value** distribution, whose pdf is:
  9 $$\begin{aligned} f(z,\xi ,\mu ,\sigma ) = \left\{ \begin{array}{ll} \exp (-\exp (-\frac{z - \mu }{\sigma }))\exp (-\frac{z - \mu }{\sigma }), &{} \text {for } \xi =0,\\ \exp (-(1-\xi \frac{z - \mu }{\sigma })^{1/c})(1-\xi \frac{z - \mu }{\sigma })^{1/c-1}, &{} \text {for } \xi \ne 0, \end{array}\right. \end{aligned}$$
  where $\xi$ is the shape parameter.
- *t*-**Student** distribution, whose pdf is:
  10 $$\begin{aligned} f(z|\nu , \mu , \sigma ) = \frac{\Gamma (\frac{\nu +1}{2})}{\Gamma (\frac{\nu }{2})\sqrt{\pi \nu }\sigma }\left( 1+ \frac{1}{\nu } \left( \frac{z-\mu }{\sigma } \right) ^2 \right) ^{-\frac{\nu + 1}{2}}, \end{aligned}$$
  where $\nu$ is the number of freedom degrees, and $\Gamma (a)$ is the gamma function defined as:
  11 $$\begin{aligned} \Gamma (a) = \int _0^{\infty } t^{a-1}e^{-t}dt, \end{aligned}$$
  where if *a* is a positive integer, then $\Gamma (a) = (a-1)!$.
- **Beta** distribution,whose pdf is defined as:
  12 $$\begin{aligned} f(z|a,b,\mu ,\sigma ) = \frac{\Gamma (a+b)((z-\mu )/\sigma )^{a-1}(1-((z-\mu )/\sigma ))^{b-1}}{\Gamma (a)\Gamma (b)}, \end{aligned}$$
  where *a* and *b* are the shape parameters.

Then, the best-fitted distribution for each voxel is determined by the minimisation of the sum of squares errors calculated as the differences between the theoretical distribution and the empirical one (histogram of the values of the groups of voxels, ${\mathbf {z}}$).

Finally, to create new instances, each pattern will be randomly generating using the estimated theoretical distribution for each of the voxels of the image.

## Dataset and experimental design

### Dataset

As we stated previously, our work is focused on SPECT 3D-images obtained after the administration of the $^{123}$I-ioflupane radiopharmaceutical, which is commonly used for binding to the presynaptic dopamine transporters in the caudate nucleus and the putamen. The substantial decrease of dopamine in the nigrostriatal dopaminergic pathway is one of the neuropathological characteristics of PD.

In this work, we have generated a dataset in collaboration with the UGC Medicina Nuclear of the Hospital Universitario ”Reina Sofía” (Córdoba, Spain). The access to the images used in this study has been done after previous anonymization by personnel authorised by the hospital. This access (previous authentication) has been registered and audited. The anonymization procedure has been carried out by means of DICOM Anonimyzer, obtaining image files that do not contain any data that could identify the patient. Each series of images was assigned a number that was related in a protected table to the origin. The entire procedure was approved and authorized by the Center’s Healthcare Administration and the UGC Medicina Nuclear. We also affirm that all methods were carried out in accordance with relevant guidelines and regulations. Finally, all subjects provided their informed consent for this study.

The data set consists of 434 studies divided according to the level of alteration of the presynaptic nigrostriatal pathway in which PD is likely to be found: 271 without alteration (class 0), 73 with a slight alteration (class 1), and 90 with severe alteration The(class 2).

**Figure 3:**
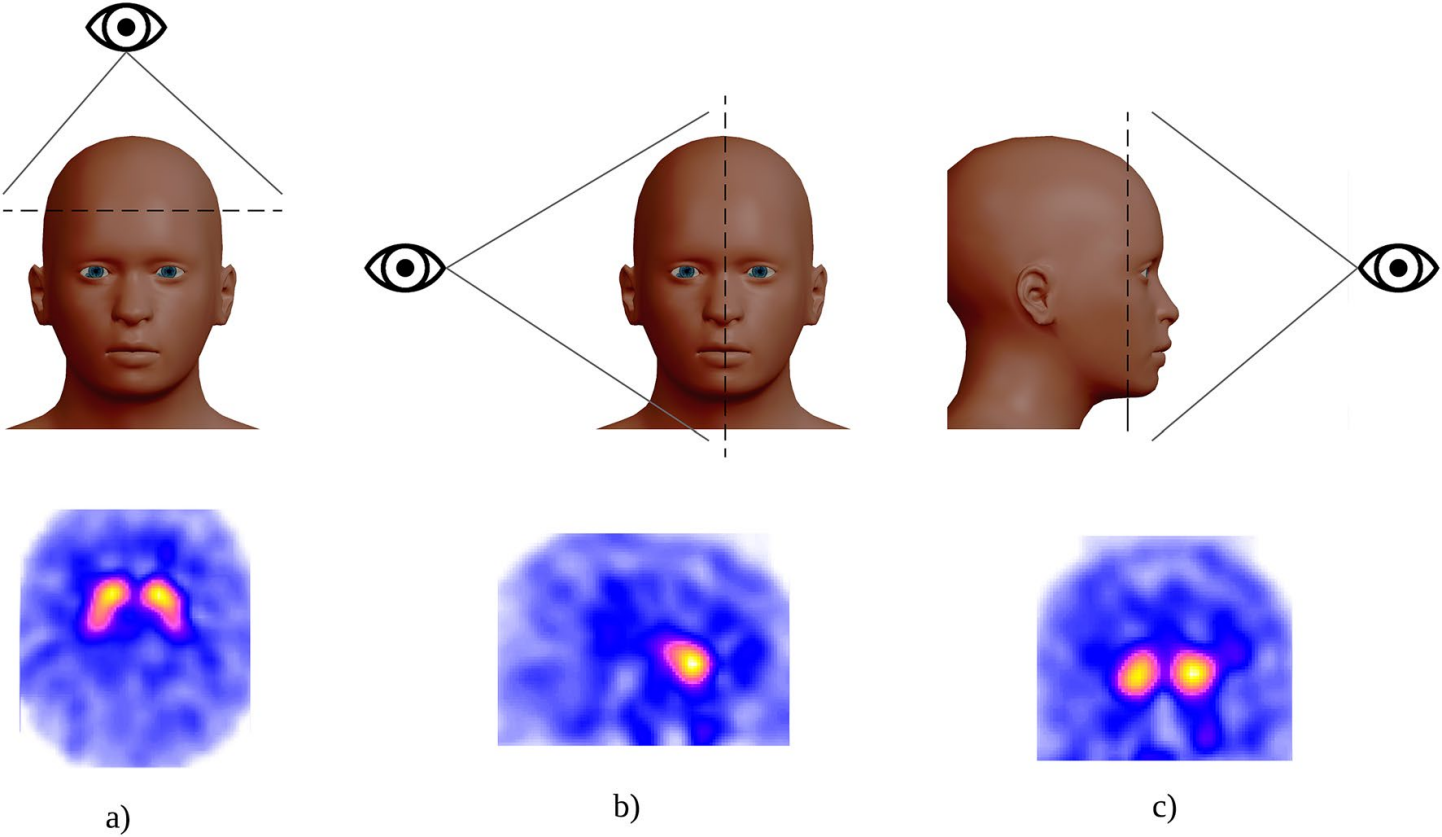
How are the cuts performed? (**a**) axial, (**b**) sagittal, and (**c**) coronal.

**Figure 4:**
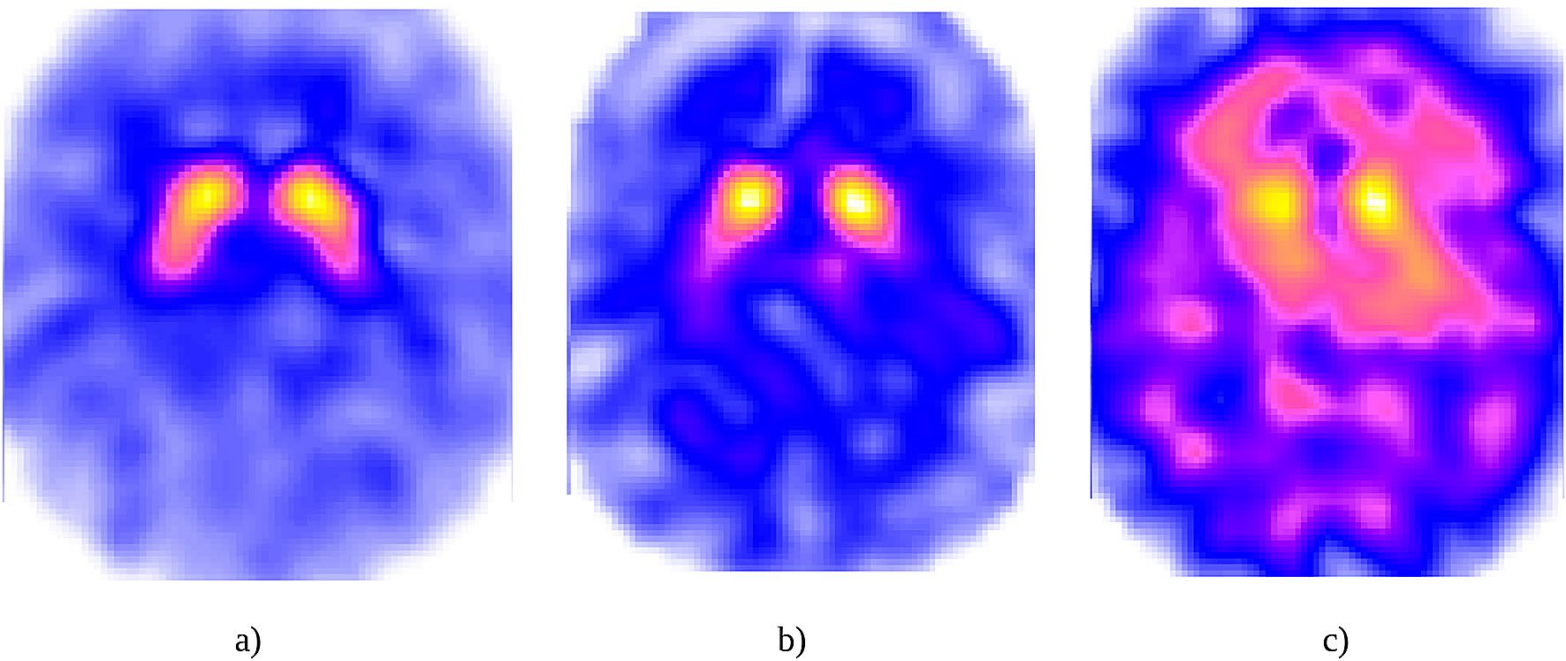
Example of a patient of each class: (**a**) without alteration (class 0), (**b**) with a slight alteration (class 1), and (**c**) with a severe alteration (class 2).

Each 3D-image has dimensionality $79\times 95\times 69$, which results in 517,845 voxels. The representation of these images can be done using different cuts in the cerebrum, which are axial, sagittal and coronal. Figure 3 shows a graphical explanation of this kind of cuts and their corresponding SPECT view. Following these representations, and considering only the axial view, Fig. 4 shows an example of a patient from each class.

### Experimental design

In “Methodology” section, we presented a methodology based on the following steps: pre-processing of 3D images, reduction of the number of features with ReliefF or OReliefF methodologies, and generation of new patterns with statistical distributions of macrovoxels. For the first step, it is not necessary to configure any parameter since the PETRA software automatically reorients the image in the $79\times 95\times 69$ dimensional space. Our main goal is to determine whether the two methods developed for feature reduction and data augmentation make sense in this type of problem using a real-world data set.

In this sense, for the second step, initially we randomly divide the database into a 70–30% stratified hold-out, that is, 70% of the images are selected for training, and the rest for test (generalisation results). The resulting training set is formed by 189 patterns of class 0, 51 patterns of class 1, and 63 of class 3. To determine if OReliefF is better than ReliefF or the original set, we perform a 5-fold cross validation over the training set (70% of the patterns as we mentioned before). We set the *k* parameter (*k*-nearest neighbours) of ReliefF and OReliefF to 5, the number of iterations *m* equal to the number of features *N*, and we explore different alternatives for the percentage of selected characteristics in the range $\{1\%,$ $2\%,$ $5\%,$ $10\%,$ $15\%,$ $20\%,$ $25\%,$ $50\%,$ $75\%\}$, considering it as a hyper-parameter to be validated. Note that the experimental validation is done using the logistic regression proposed by Rennie and Srebro^46^.

Once the best feature selector is determined, we use the reduced dataset to continue for the third step, where we test the proposed method for data augmentation, based on the use of different statistical distributions. Firstly, we estimated the best statistical distribution for each voxel (considering all voxels of its macrovoxel). Then, we consider different configurations to generate new brains for the training set:

- CONF0: Without applying data augmentation.
- CONF1: Duplicate the patterns of each class.
- CONF2: Duplicate the patterns of the minority classes 1 and 2 (subjects with slight or severe alteration).
- CONF3: Duplicate the patterns of the minority class 1 (subjects with slight alteration).
- CONF4: Duplicate the patterns of the second minority class 2 (subjects with severe alteration).
- CONF5: Triplicate the patterns of the minority class 1 (subjects with slight alteration).

These five configurations are compared against the simple method of noisy replication^40^ (referrered to as RAND in this paper), where we randomly choose patterns from the class to be oversampled and replicate them including a small amount of noise. For this RAND configuration, we have considered a normal distribution with mean zero and a standard deviation of 0.01 (*N*(0, 0.01)). Moreover, the configuration used is triplicating class 1, subjects with slight alteration, because, as above discussed, this is the one leading to the best results.

Finally, we executed the procedure of data augmentation 30 times given the stochasticity in the procedure of the generation, and we compared the results obtained in the test set (30% of the initial hold-out) to determine which configuration is the best in our problem. To check the results obtained, we plot informative voxels found in the brain by our algorithm, showing that they are not limited to the classical ROIs.

## Results and discussion

### Results analysis

To analyse the validity of the proposed OReliefF algorithm, Table 1 shows the results in CCR and MMAE with the different configurations proposed in the previous section, that is, using 5-fold cross-validation over the training set to determine the best parameter setting. These validation results help establish the most appropriate percentage of voxels to be considered during classification, together with the best alternative for ReliefF (nominal or ordinal), based on which, we will evaluate the test results.

**Table 1:** OReliefF versus ReliefF comparison in terms of *CCR* and *MMAE* obtained by the ordinal classifier without the application of data augmentation (5-fold cross validation results).

ReliefF	*MMAE* ($\downarrow$)	*CCR* ($\uparrow$)
1%	0.6470	0.7426
2%	0.7255	0.7129
5%	0.6471	0.7393
10%	0.5490	**0.7756**
15%	0.5686	0.7591
20%	*0.5238*	0.7624
25%	0.5556	0.7525
50%	*0.5238*	0.7525
75%	*0.5238*	0.7690

OReliefF	*MMAE* ($\downarrow$)	*CCR* ($\uparrow$)
1%	0.5882	*0.7723*
2%	0.7059	0.7261
5%	0.6471	0.7558
10%	0.5882	0.7624
15%	**0.5098**	*0.7723*
20%	*0.5238*	0.7525
25%	0.5556	0.7558
50%	0.5556	0.7558
75%	0.5397	0.7624

No ReliefF	*MMAE* ($\downarrow$)	*CCR* ($\uparrow$)
100%	0.5397	0.7657

The best results are shown in bold. The second best results are shown in italics

As can be seen, the best feature selector in terms of *CCR* is the ReliefF when we maintain 10% of the features for the classifier, but its *MMAE* is not good. Thus, the second best algorithm in terms of *CCR* is the proposed OReliefF when 15% of the characteristics are used for the classifier, and, in addition, it is the best configuration in terms of *MMAE*. We can affirm that OReliefF results in a better feature selection, because the *CCR* only drops from 0.7756 to 0.7723 which is practically the same value, while the *MMAE* improves from 0.5490 to 0.5098. If we do not apply any feature selection the results are 0.5397 in *MMAE* and 0.7657 in *CCR*, which are worse than the obtained by the proposed methodology.

**Figure 5:**
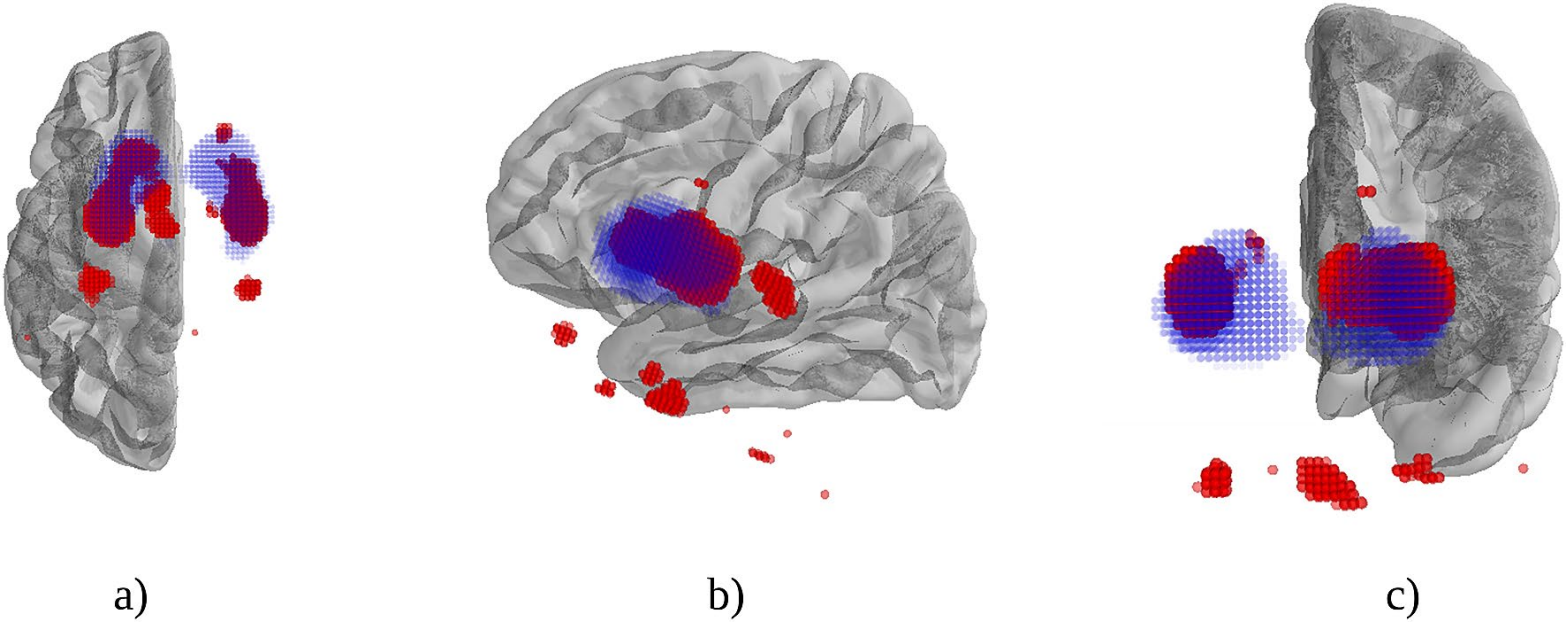
Example of informative voxels outside (red points) the ROIs areas (blue points) in a patient showing (**a**) axial, (**b**) sagittal and (**c**) coronal views.

Given the above results, we consider the reduction of the dataset with a 15% of the characteristics (informative voxels) returned by the proposed OReliefF algorithm. In this sense, for each 3D-image, a total of 517,845 voxels are reduced to 77,676 informative voxels. The selected voxels have been graphically represented in red in Fig. 5, together with the ROIs corresponding to caudate and putamen in blue, which are the common areas considered for PD diagnosis. A video has also been uploaded in the website of the research group (http://www.uco.es/ayrna/parkinson/informativeVoxels.mp4), where the image is rotated to ease visualisation. As can be checked, there are distinct informative voxels outside the classical ROIs. They correspond to small cortical areas, predominantly temporal and in the medial region of the parietal lobe. These extrastriatal areas follow a recent tendency of other studies (e.g. magnetic resonance imaging) which show alterations in brain areas outside the striatum. This opens up a much deeper area of investigation for future work, as these zones are difficult to identify anatomically.

The next step is the generation of new patterns according to the data augmentation procedure described in “Data augmentation” section with the configurations shown in “Experimental design” section. We compare the results against the noisy replication strategy (RAND strategy also described in “Experimental design” section). It is important to mention that the generation is done using only the selected informative voxels so that the macrovoxels can have empty voxels, but it is not a problem because they provide enough information to estimate the theoretical probability distribution.

**Table 2:** Mean and standard deviation results in terms of *CCR* and *MMAE* for the 30 executions of the data augmentation algorithm (test set).

Configuration	*MMAE* ($\downarrow$)	*CCR* ($\uparrow$)
CONF0	0.6364	**0.7710**
CONF1	$0.6303 \pm 0.0155$	$0.7588 \pm 0.0054$
CONF2	$0.6349 \pm 0.0082$	$0.7575 \pm 0.0051$
CONF3	$0.6212 \pm 0.0214$	$0.7646 \pm 0.0053$
CONF4	$0.6364 \pm 0.0000$	$0.7557 \pm 0.0000$
CONF5	**0.5561 ± 0.0192**	*0.7685 ± 0.0036*
RAND	*0.5958 ± 0.0208*	0.7613 ± 0.0048

The best results are shown in bold. The second best results are shown in italics

The results in Table 2 show the performance of the classifier when we use the proposed data augmentation and compare it against the noisy replication method. For the reasons presented in the “Methodology” section, *CCR* and *MMAE* are conflicting objectives. In this way, for an imbalanced ordinal classification problem, optimising the *MMAE* metric, without significantly reducing the value of *CCR*, should be considered as a priority. The configuration number 0, which corresponds to the reduced data set without the application of data augmentation, has the best *CCR* but its *MMAE* is the worst, showing that the algorithm tend to classify all the patterns into the majority class (subjects without alteration) to the detriment of the minority classes (subjects with slight or severe alteration). In almost all cases, when data augmentation is applied, the classifier improves the results in *MMAE* showing the ability of the proposed method to give more importance to subjects with alteration without losing *CCR* quality.

Looking at the different configurations, it seems that the generation of patterns for the classes corresponding to subjects with alteration involves a high decrease of the *MMAE* from 0.6364 to 0.6212 when we double the patterns and up to 0.5561 when we triple them. In addition, the *CCR* only decreases from 0.7710 to 0.7685, which is negligible considering the large improvement in the *MMAE* metric. Thus, CONF5 is the best configuration given that a much lower *MMAE* value, $0.5561 \pm 0.0192$, is obtained when compared to the other configurations, without losing too much accuracy (*CCR* value is $0.7685 \pm 0.0036$, the second best average value). It makes sense because class 1 (the oversampled class in this configuration, individuals with slight alteration) is the most difficult one to be correctly classified. Other configurations also improve the quality of the error metric, but we consider that it is caused by the pattern generation in class 1. Finally, when compared against the noisy replication method (RAND configuration, where we also triplicate the slight alteration class), the results in *CCR* are very similar, but CONF5 is able to significantly improve the levels of *MMAE*. In this way, the use of the fitted statistical distribution of the macrovoxel structures help generate new patterns that better respect the ordinal disposition of the minority class.

In order to check whether the differences found in Table 2 could be attributed to randomness, we apply a set of statistical tests. First of all, we need to assess the character of the results obtained regarding their distribution: if they follow a normal distribution, we will apply parametric tests, while non-parametric tests will be considered in other case. We discard the result for CONF0, given that, as only one test value is available, it does not make sense to apply statistical tests, the *MMAE* being clearly worse than those obtained by data augmentation.

First, we analyse the *MMAE* results. One Kolmogorov–Smirnov (K–S) test^54^ is used for each set of 30 results, rejecting all the hypotheses of normality (*p* values $<0.001$), i.e. non-parametric tests need to be applied. Consequently, we select the Kruskal–Wallis (K–W) test^55^, which is a non-parametric alternative for the one-way analysis of variance, when we want to compare more than two independent samples. The results of this test are included in Table 3. This table includes the average ranks obtained for each method, where the lower the rank, the better the method (as *MMAE* has to be minimised). It also includes the associated $\chi ^2$ and the corresponding *p* value, showing that there are significant differences in test *MMAE*, where the two best configurations (according to the ranks) are CONF5 and RAND. These two configurations obtain the best results because both are using the option of triplicating patterns from class 1 (slight alteration), which seems to be the most problematic in this problem (easily confused with neighbouring classes). In order to establish the significance of the differences between CONF5 and RAND for test *MMAE* results, we finally apply the Mann–Whitney (M–W) test^56^, a post-hoc non-parametric test for independent samples, which results in a *p* value$<0.001$, i.e. there are significant differences favouring CONF5 with respect to RAND.

**Table 3:** Kruskal–Wallis statistical test results for *CCR* and *MMAE* (test set).

Configuration	Average ranks	
	*MMAE*($\downarrow$)	*CCR* ($\uparrow$)
CONF1	115.17	71.60
CONF2	122.92	59.93
CONF3	99.67	$120.25$
CONF4	125.50	47.00
CONF5	$\mathbf {20.78}$	$\mathbf {146.50}$
RAND	$58.97$	97.72
$\chi ^2$	129.33	95.38
*p* value	$<0.001$	$<0.001$

The best results are shown in bold. The second best results are shown in italics

If we repeat the same statistical treatment with the test *CCR* results, the K–S test again rejects the hypotheses of normality for all the results (*p* values$<0.001$). The results of the M–W test for the test *CCR* values are also included in Table 3, showing that there are significant differences and that the two best configurations are, in this case, CONF5 and CONF3. The M–W establishes again significant differences favouring CONF5, with a *p* value of 0.004.

Summarising, CONF5 applied to the data augmentation process proposed in this paper stands out as a new way to generate synthetic brain patterns that help the classification of the most confusing class (slight alteration). It can be a decision support system for medical purposes where, not only the global correct classification of patients is important, but also a good accuracy in the confusing class corresponding to slight level of alteration of the presynaptic pathway. In this way, this work suggests a new option to approach at PD, with good values for diagnosis accuracy and classification in the different disease stages.

## Conclusions

In this paper, we propose a new methodology for classifying different stages of PD using an ordinal classifier from 3D-images obtained after the administration of ^123^I-ioflupane in three classes depending on the level of affectation of the image. The classes corresponds to patients without alteration of the presynaptic nigrostriatal pathway, with a slight alteration, or with a severe alteration. This methodology is based on an ordinal logistic regression model where we have previously executed an ordinal filter algorithm to select the most discriminant voxels and a novel data augmentation technique based on probability distributions associated with samples formed by macrovoxels to classify better the classes corresponding to subjects with alteration. In this difficult problem, where it is sometimes necessary the concurrence of several experts to determine the value of the label associated with the 3D-image, we have managed to decrease the metric related to the good classification of the intermediate class, the most difficult to diagnose, by 12.62%, still obtaining a very acceptable overall classification, an average of 76.85% and a standard deviation of 0.36%. It is important to note that both the ordinal feature selection method and the data augmentation method could be applied to other datasets and could be coupled with other classifiers. Finally, we would like to point out that the new database on which we have carried out the validation is very interesting for this type of study, and we will make it available to the scientific community in order to carry out experiments with it and make comparisons with our methodology.

Regarding the limitations of the present study, the size of the dataset could still be increased, by including more subjects, what would better assess the generalisation capability of the proposals. Another problem associated with the method proposed lies in the fact that the computational cost of feature selection process is high, given that all the areas of the brain are considered for classification (although this enables the classifier to find new informative voxels, not previously considered in the literature). Given that a definitive diagnosis is not always available, the models depend on a clinical evaluation (from the labels in the dataset), which unavoidably includes certain degree of subjectivity and can influence the training process (i.e. classification accuracy may be affected by variations in clinical confidence). Another limitation may be derived from the main clinical objective: the automatic separation between control subjects and parkinsonian patients with different degrees of affectation. However, the most challenging objective in PD diagnosis is obtaining a method able to differentiate between the different parkinsonian syndromes.
